# Photosynthesis in the near infrared: the γ subunit of *Blastochloris viridis* LH1 red-shifts absorption beyond 1000 nm [Author-notes an1]

**DOI:** 10.1042/BCJ20220585

**Published:** 2023-03-29

**Authors:** Andrew Hitchcock, David J.K. Swainsbury, C. Neil Hunter

**Affiliations:** 1 Plants, Photosynthesis and Soil, School of Biosciences, University of Sheffield, Sheffield S10 2TN, U.K.; 2 School of Biological Sciences, University of East Anglia, Norwich NR4 7TU, U.K.

## Abstract

The reaction centre (RC) in purple phototrophic bacteria is encircled by the primary light-harvesting complex 1 (LH1) antenna, forming the RC–LH1 ‘core’ complex. The Q_y_ absorption maximum of LH1 complexes ranges from ∼875–960 nm in bacteriochlorophyll (BChl) *a-*utilising organisms, to 1018 nm in the BChl *b-*containing complex from *Blastochloris* (*Blc.*) *viridis*. The red-shifted absorption of the *Blc. viridis* LH1 was predicted to be due in part to the presence of the γ subunit unique to *Blastochloris* spp., which binds to the exterior of the complex and is proposed to increase packing and excitonic coupling of the BChl pigments. The study by Namoon et al. provides experimental evidence for the red-shifting role of the γ subunit and an evolutionary rationale for its incorporation into LH1. The authors show that cells producing RC–LH1 lacking the γ subunit absorb maximally at 972 nm, 46 nm to the blue of the wild-type organism. Wavelengths in the 900–1000 nm region of the solar spectrum transmit poorly through water, thus γ shifts absorption of LH1 to a region where photons have lower energy but are more abundant. Complementation of the mutant with a divergent copy of LH1γ resulted in an intermediate red shift, revealing the possibility of tuning LH1 absorption using engineered variants of this subunit. These findings provide new insights into photosynthesis in the lowest energy phototrophs and how the absorption properties of light-harvesting complexes are modified by the recruitment of additional subunits.

The photosynthetic reaction centre (RC) from the anoxygenic phototrophic bacterium *Blastochloris* (*Blc*.) *viridis* has historical significance as the first membrane protein to have its structure determined by X-ray crystallography, resulting in the award of the Nobel Prize in Chemistry to Johann Deisenhofer, Robert Huber and Hartmut Michel in 1988 [1,2]. The *Blc. viridis* RC consists of H, M, L and C subunits and, as in other phototropic purple bacteria, is surrounded by a core light-harvesting complex 1 (LH1) antenna forming the RC–LH1 ‘core’ complex. The role of the LH1 antenna is to increase light-harvesting capacity and transfer excitation energy to the RC, inducing photochemical charge separation originating from the ‘special pair’ of bacteriochlorophyll (BChl) pigments leading to the generation of a quinol. Quinols leave the RC via a gap in the LH1 ring and are re-oxidised at the cytochrome *bc*_1_ complex, generating a proton-motive force to drive ATP synthesis.

In 2018, the 2.9 Å resolution structure of the *Blc. viridis* core complex [3] was the first in the new wave of structures of photosynthetic RC and light-harvesting complexes brought about by significant advances in cryogenic electron microscopy (cryo-EM). The structure revealed that the *Blc. viridis* LH1 consists of 17 heterodimers of α and β polypeptides and 16 copies of the γ subunit (Figure 1), which is uniquely found in *Blastochloris* species and packs between the β subunits on the exterior of the LH1 ring. The ‘break’ in the LH1 ring due to the absence of a 17^th^ γ subunit creates a channel for quinone/quinol exchange between the RC and the cytochrome *bc*_1_ complex (Figure 1). LH1 contains 14–16 αβ pairs in other phototrophic purple bacteria (see references [4–11] for examples), where it may fully encircle the RC, as in *Blc. viridis*, limiting quinone traffic to small gaps in the LH1 ring, or be held open by the presence of additional subunits that create larger and more efficient routes for quinone/quinol diffusion. Unlike the *Blc. viridis* γ subunit, these LH1-ring-interrupting subunits are typically present in a single copy per complex, rather than in a near-stoichiometric arrangement with the α and β polypeptides.

**Figure 1.**
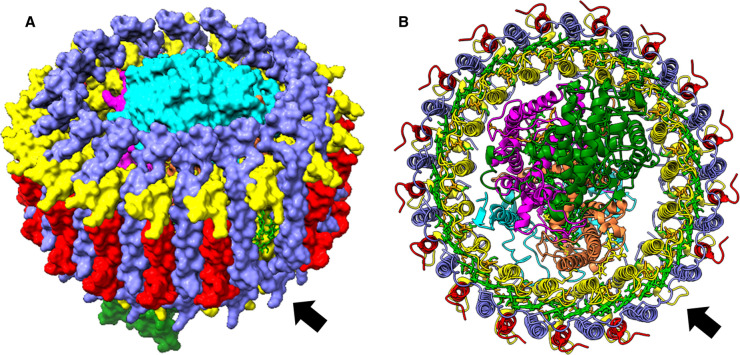
Structure of the *Blc. viridis* RC–LH1 complex. (**A**) Tilted view with the protein in surface representation. (**B**) View looking down at the periplasmic face of the complex with the protein in cartoon representation. The RC subunits are in cyan (RC-H), magenta (RC-M), orange (RC-L) and green (RC-C). LH1α and β are in yellow and purple, respectively, and LH1γ is in red. BChls are shown in green and quinones in yellow. The gap in the LH1 ring due to the absence of a 17th γ subunit is visible on the front right of the complex, as indicated by the black arrows.

Each LH1 αβ subunit co-ordinates a pair of excitonically coupled BChls and one carotenoid (with the current exception of the *Rhodobacter* (*Rba.*) *sphaeroides and Rba. capsulatus* complexes, which have two carotenoids per αβ pair [10,11]); thus the 17-mer *Blc. viridis* LH1 binds 34 BChls and 17 carotenoids [3]. Unlike most anoxygenic photosynthetic bacteria, which utilise BChl *a* for light harvesting and charge separation*, Blc. virdis* uses BChl *b* as its major photosynthetic pigment. The *Blc. viridis* RC–LH1 absorption, with a Q_y_ maximum at 1018 nm in whole cells or 1008 nm when solubilised in detergent, is considerably red-shifted compared with BChl *a*-containing complexes, most of which absorb maximally at ∼875–880 nm, although some bind calcium ions that shift absorption beyond 900 nm [4,12] and as far as 963 nm in *Thiorhodovibrio* strain 970 [9]. These adaptations for utilising longer wavelengths of light contrast with those adopted by cyanobacteria, where chlorophyll (Chl)-based photosynthesis in far-red light is achieved using modified RC and antenna complexes that bind Chls *d* and *f*, often in a reversible acclimation process in response to far-red illumination [13].

It has previously been suggested that the red shift of *Blc. viridis* LH1 beyond 1000 nm is due to a combination of incorporation of BChl *b* rather than BChl *a*, the presence of the additional LH1 αβ pair, and the binding of the unique γ subunit, which is proposed to enhance structural stability and tighten the packing of BChls around the LH1 ring to increase the excitonic coupling between them [3]. Now, elegant work by Namoon, Rudling and Canniffe [14] has determined the effect of the absence of the γ polypeptide using engineered strains of *Blc. viridis*, providing *in vivo* experimental evidence for these functional roles for the first time.

When grown with illumination from halogen bulbs (broad emission from ∼350 nm to ∼850 nm), the authors’ standard choice for *Blc. viridis*, LH1 was not assembled in mutants lacking LH1γ*,* which grew more slowly than the wild type and had whole cell absorption maxima at 825 nm, corresponding to free RCs. Conversely, growth with illumination from fluorescent lamps, which do not emit beyond ∼650 nm, did result in properly assembled RC–LH1 complexes in the ΔLH1γ strain, which had an absorption maximum at 972 nm, blue-shifted by 46 nm compared with the wild-type cells at 1018 nm (Figure 2). A major pigmented band slightly less dense/smaller than that present in the wild type was isolated from detergent-solubilised membranes from the mutant, corresponding to RC–LH1 complexes lacking the γ subunit; the isolated γ-free RC–LH1 complex absorbed maximally at 965 nm, blue-shifted by 43 nm compared with the wild-type complex at 1008 nm. By red-shifting absorption of the complex past the 900–1000 nm region of the solar spectrum, where sunlight transmits poorly through water vapour in the atmosphere and liquid water at the Earth's surface, LH1γ gives *Blc. viridis* access to a region of the spectrum where photons are more abundant, providing a rationale for the recruitment of LH1γ during the evolution of the lowest energy photosynthetic antenna-RC complex. Further work is required to understand why the γ-free LH1 complex accumulated in cells grown under white light but not during growth with illumination from halogen bulbs, which may uncover hitherto unknown light-dependent regulation of core complex assembly in *Blc. viridis*. The authors also highlight the increasing difficulty in obtaining light bulbs that emit in the far-red-near-infrared region of the spectrum. This may appear trivial but represents a significant challenge for culturing anoxygenic phototrophs under physiologically relevant light regimes and will also limit our ability to isolate phototrophs that operate at similar or lower energy than *Blastochloris* species, which may already exist in nature.

**Figure 2.**
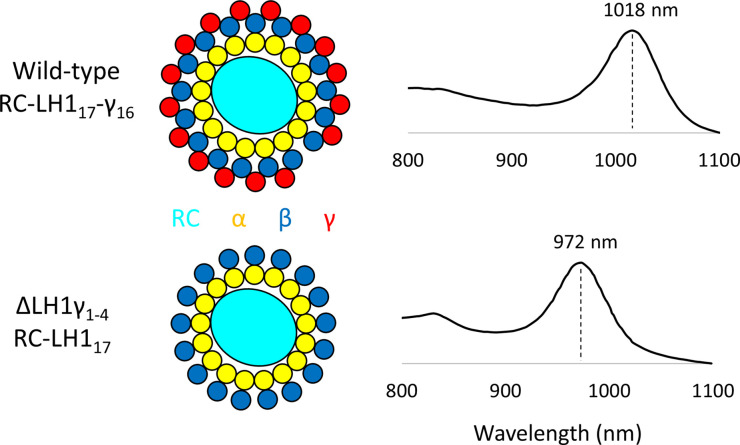
Schematic representation of wild-type and ΔLH1γ *Blc. viridis* RC–LH1 complexes and the Q_y_ maxima of the whole cell absorption spectra of the corresponding strains. The absence of the γ subunit results in a blue-shifted Q_y_ absorption at 972 nm, compared with 1018 nm in the wild type. Note that the schematic of the complex for the mutant strain is a prediction as this structure has not been determined. The RC is represented in cyan, LH1α in yellow, LH1β in blue and LH1γ in red.

Bands corresponding to RCs and free pigments were also present in sucrose gradients from the ΔLH1γ mutant grown under white light, suggesting that a proportion of complexes fails to assemble an LH1 antenna and/or that the LH1 complex is less stable in the absence of the γ subunit, in keeping with the idea that γ plays a role in complex stability. It will be interesting to see how stable the purified γ-less core complex is compared with wild-type RC–LH1, and, if it is sufficiently stable to permit structural studies, how the absence of γ affects the packing of the LH1 BChls. The intra-subunit (8.8 Å) and inter-subunit (8.5 Å) Mg–Mg distances in the wild-type *Blc. virids* LH1 complex are the shortest reported for any LH1 ring [3]; presumably these distances are increased in the absence of γ, explaining the blue-shifted absorption compared with the wild-type complex. Purification of the γ-less RC–LH1 complex would also permit measurement of the rate of excitation energy transfer (EET) from LH1 to the RC. The BChl *b* special pair in *Blc. viridis* absorbs at 960 nm, making the transfer from LH1 ‘uphill’ by ∼58 nm in the wild-type core complex, but only ∼12 nm in the mutant complex; uphill EET is common in purple bacterial RC–LH1 complexes and is driven by thermal energy from the environment. Therefore, assuming the distances are the same, one would predict that the rate of LH1-to-RC EET would be faster for the mutant due to increased spectral overlap and the lowered energy gap between the LH1 and RC pigments. Furthermore, a complex lacking γ presumably has 17 potential quinone import/quinol export sites and diffusion of these molecules across the barrier formed by the LH1 ring is expected to be accelerated compared with the wild-type complex, where quinone/quinol exchange is limited to the single gap in LH1 created by the ‘missing’ γ subunit.

*Blc. viridis* contains four putative LH1γ polypeptides; LH1γ_1–3_ are very similar and are encoded next to one another in the genome, and all three can be incorporated into LH1 [15]. Conversely, the more divergent LH1γ_4_ has not been identified in *Blc. viridis* RC–LH1 preparations; consistent with this, Namoon et al. found no difference in the RC–LH1 complexes produced in their ΔLH1γ_1–3_ and ΔLH1γ_1–4_ strains. Further work is needed to address how the LH1γ_1–3_ genes are regulated to ensure co-ordinated expression with *pufA* and *pufB* and correct incorporation of γ into LH1. These are considerations not limited to the *Blc. viridis* core complex; other than PufX, which is encoded in an operon with the *pufBA* genes that encode the LH1 α and β polypeptides in the photosynthesis gene cluster (PGC) in *Rhodobacter* species [16], a common theme with ‘additional’ LH1 subunits is that they tend to be encoded at distinct genomic locations to the genes encoding the RC and LH1 polypeptides. For example, protein W in *Rhodopseudomonas* (*Rps.*) *palustris,* proteins Y and Z in *Rba. sphaeroides,* and the *Blc. viridis* γ subunits are all encoded outside of their respective PGCs [5,10,14,17–18].

Despite the apparent absence of production under standard laboratory growth conditions, Namoon et al. show that plasmid-driven production of LH1γ_4_ in the ΔLH1γ background resulted in the accumulation of RC–LH1 complexes with a whole cell absorption maximum at 1003 nm, 15 nm shorter than that of the wild type or the mutant complemented with LH1γ_1_. Thus, the absorption properties of LH1 appear to be modified by incorporation of different LH1γ subunits, leading the authors to suggest that engineered variants of LH1γ may allow fine-tuning of LH1 absorption and increased red-shifting towards the theoretical low-energy limit of ∼1100 nm, where there is sharp drop in the solar radiation that reaches the Earth's surface [14]. A 1100-nm absorbing LH1 would have to transfer energy 140 nm uphill to the *Blc. viridis* RC special pair; this is greater than the ∼90 nm uphill energy transfer in *Thiorhodovibrio* strain 970 [9], which represents the maximum energy gap reported in natural RC–LH1 systems, and it will be fascinating to discover the longest wavelength photons that can drive charge separation should the authors succeed in pushing LH1 absorption beyond 1018 nm.

Recently, an extra LH1 subunit referred to as γ-like was found in the structure of the core complex from the extremophilic phototroph *Rhodopila* (*Rpi.*) *globiformis* [19]. Eleven copies of this single transmembrane helix protein are located between the β-polypeptides in the 16 subunit *Rpi. globiformis* LH1 ring, with cardiolipins present in the remaining five sites. These γ-like proteins form hydrophobic interactions with the β subunits and, like the *Blc. viridis* LH1γ, are proposed to have a stabilising function, as well as occluding quinone transport through the LH1 subunits to which they bind. However, the absorption maximum of the *Rpi*. *globiformis* complex at 879 nm does not display a significant red shift compared with the 875–880 nm peak of typical LH1 complexes from BChl *a*-utilising organisms, thus these newly identified γ-like subunits do not appear to have a similar red-shifting role to the *Blc. viridis* γ subunit, consistent with their low sequence identity and differences in their interactions with the LH1 ring. A second recent study also identified a novel light-harvesting complex subunit referred to as γ, this time in the peripheral LH2 antenna complexes of *Rps. palustris* [20]. Repeats within this protein form an extended undulating ribbon around six of the nine LH2 αβ heterodimers, breaking the symmetry of the nonameric LH2 complex and coordinating six additional BChls, enhancing absorption at 800 nm. The structure and function of this protein are thus distinct from those of the *Blc. viridis* LH1γ subunit.

In summary, the paper by Namoon, Rudling and Canniffe provides clear experimental demonstration of red-shifting of *Blc. viridis* LH1 absorption by the γ subunit. In the absence of γ, LH1 absorption falls into a region of the solar spectrum where photon abundance at the Earth's surface is low and transmission through water is poor, providing an evolutionary rationale for recruitment of the γ-polypeptide to the complex. This work paves the way for structural and spectroscopic characterisation of the γ-free RC–LH1 complex, as well as the possibility of generating LH1 antennae with a range of Q_y_ absorption maxima by incorporation of modified LH1γ subunits. If the authors can push LH1 absorption further into the near infrared creating new lower-energy antenna complexes, this will provide a route to determine the low-energy limit of photosynthesis. It will also demonstrate rational tuning of absorption of an antenna complex, which has wider implications for reimagining the capture of light by natural and artifical photosynthetic systems.

## Abbreviations

BChl: bacteriochlorophyll
*Blc.*: *Blastochloris*
Chl: chlorophyll
cryo-EM: cryogenic electron microscopy
EET: excitation energy transfer
LH1: light-harvesting complex 1
LH2: light-harvesting complex 2
PGC: photosynthesis gene cluster
*Rba.*: *Rhodobacter*
RC: reaction centre
*Rpi.*: *Rhodopila*
*Rps.*: *Rhodopseudomonas*
